# Correction: Neuronal calmodulin levels are controlled by CAMTA transcription factors

**DOI:** 10.7554/eLife.104114

**Published:** 2024-10-21

**Authors:** Thanh Thi Vuong-Brender, Sean Flynn, Yvonne Vallis, Saliha E Sönmez, Mario de Bono

**Keywords:** *C. elegans*

Vuong-Brender TT, Flynn S, Vallis Y, Sönmez SE, de Bono M. 2021. Neuronal calmodulin levels are controlled by CAMTA transcription factors. *eLife* **10**:e68238. doi: 10.7554/eLife.68238.

Published 9 September 2021

In this paper we show that the CAMTA transcription factor regulates neuronal levels of calmodulin in *C. elegans* and in *Drosophila*. In revising the paper for publication, we imaged *Drosophila* retinae by immunofluorescence and showed that calmodulin levels were reduced in photoreceptors of Camta mutants compared to control flies. These results are shown in Figure 5D, E and Figure 5—figure supplement 1. S. Ece Sönmez’ expertise with dissecting, staining and mounting fly retinae was invaluable in acquiring the data. We are therefore formally correcting the eLife paper to include S. Ece Sönmez on this paper. S. Ece Sönmez has reviewed the manuscript and supports the conclusions, and agrees to be responsible for all parts of the paper in its published form.

Details of S. Ece Sönmez’s contribution are explained below:

S. Ece Sönmez contributed to the revision experiments of this manuscript, in particular expert preparation of some of the samples from which the data presented in Figure 5D, E and Figure 5—figure supplement 1, involving immunofluorescence imaging of *Drosophila* retinae, were acquired.

**New authors list:**

Thanh Thi Vuong-Brender, Sean Flynn, Yvonne Vallis, Saliha E Sönmez, Mario de Bono

**Original authors list:**

Thanh Thi Vuong-Brender, Sean Flynn, Yvonne Vallis, Mario de Bono

**Details for the omitted author:**

Saliha E Sönmez

Institute of Science and Technology Austria (IST Austria), Klosterneuburg, Austria

**Contribution:** Investigation

**Competing interests:** No competing interests declared

The following sentence from the Acknowledgement has been removed:

The authors are very grateful to Salihah Ece Sönmez for teaching us how to dissect, mount and stain *Drosophila* retinae.

The article has been corrected accordingly.

